# Large Vessel Disease Modifies the Relationship Between Kidney Injury and Cerebral Small Vessel Disease

**DOI:** 10.3389/fneur.2018.00498

**Published:** 2018-06-26

**Authors:** Dong-Hui Ao, Fei-Fei Zhai, Fei Han, Li-Xin Zhou, Jun Ni, Ming Yao, Ding-Ding Zhang, Ming-Li Li, Xiao-Hong Fan, Zheng-Yu Jin, Li-Ying Cui, Shu-Yang Zhang, Yi-Cheng Zhu

**Affiliations:** ^1^Department of Neurology, Peking Union Medical College Hospital, Chinese Academy of Medical Sciences and Peking Union Medical College, Beijing, China; ^2^Central Research Laboratory, Peking Union Medical College Hospital, Chinese Academy of Medical Sciences and Peking Union Medical College, Beijing, China; ^3^Department of Radiology, Peking Union Medical College Hospital, Chinese Academy of Medical Sciences and Peking Union Medical College, Beijing, China; ^4^Department of Nephrology, Peking Union Medical College Hospital, Chinese Academy of Medical Sciences and Peking Union Medical College, Beijing, China; ^5^Department of Cardiology, Peking Union Medical College Hospital, Chinese Academy of Medical Sciences and Peking Union Medical College, Beijing, China

**Keywords:** large artery atherosclerosis, artery stiffness, kidney injury, cerebral small vessel disease, interaction

## Abstract

**Background:** Recent studies have shown that renal disease is associated with magnetic resonance imaging (MRI) markers of cerebral small vessel disease (CSVD), independent of traditional vascular risk factors. Although large artery lesions might be involved in the cerebrorenal association, evidence has been lacking.

**Methods:** A total of 928 participants from a population-based cohort study were included. Kidney injury measurements included urinary albumin-to-creatinine ratio (ACR) and estimated glomerular filtration rate (eGFR). CSVD was assessed on MRI by white matter hyperintensity volume (WMHV), lacunes, brain parenchymal fraction (BPF), cerebral microbleeds (CMBs), and perivascular space. Carotid plaques and brachial-ankle pulse wave velocity (baPWV) were used to assess large artery atherosclerosis and stiffness. Multivariable linear and logistic regression and additional interaction models were used for statistical analysis.

**Results:** Individuals with elevated ACR had higher prevalence of lacunes and more WMHV (*p* = 0.001 and 0.000, respectively), those with decreased eGFR had smaller brain volume, higher prevalence of lacunes and deep CMBs (*p* = 0.009, *p* = 0.017) and *p* = 0.010 respectively). Interaction analysis revealed that carotid plaque and baPWV significantly enhanced the association between eGFR and BPF (*p* = 0.001 and *p* = 0.002, respectively), that is, the association of eGFR with BPF was only significant among participants with carotid plaque and higher baPWV. In addition, carotid plaque enhanced the association between ACR and WMHV (*p* = 0.034) and baPWV enhanced the association between ACR and the presence of lacunes (*p* = 0.027). Modifying effect of large vessel disease markers on the association between kidney injury measurements and CMBs was not significant.

**Conclusion:** Evaluation of subclinical CVSD in individuals with kidney injury is warranted, especially in those with combined large artery disease.

## Introduction

Both chronic kidney disease and cerebral small vessel disease (CSVD) are highly prevalent in the elderly population (1, 2). Previous studies have demonstrated the association between deterioration of kidney injury measurements and magnetic resonance imaging (MRI) markers of CSVD (3–7). Since this association could not be fully explained by shared traditional vascular risk factors, several hypotheses on mechanisms underlying cerebrorenal association were raised. For example, inflammatory processes can trigger both kidney and brain vascular injury and also play a role in endothelial dysfunction. Moreover, as renal disease deteriorates, uremic toxins, sodium and water retention, and abnormal calcium and phosphate metabolism additionally lead to cerebrovascular damage (8, 9).

Theoretically, large artery lesions might also be involved in the cerebrorenal interaction. First, both kidney and brain are low vascular-resistance systems, allowing continuous high-volume perfusion; hence, arterioles of these 2 organs might be similarly susceptible to the resilient change of proximal large artery lesions (10). In addition, severity of large artery atherosclerosis indicates burdens of internal vascular risks that may simultaneously lead to kidney injury and CSVD. Indeed, different studies reported that large artery lesions (e.g., atherosclerosis or stiffness) are associated with renal disease (11, 12) or CSVD imaging markers, respectively (13, 14). However, whether the presence of large artery lesions, manifested as atherosclerosis or aortic stiffness, would modify the association between renal disease and CSVD remains largely unknown.

We investigated the association of kidney injury measured by urinary albumin-to-creatinine ratio (ACR) and estimated glomerular filtration rate (eGFR) with CSVD MRI markers in a community-dwelling population. Artery atherosclerosis as well as aorta stiffness were assessed in order to delineate large vessel lesions. We hypothesized that cerebrorenal association may differ between groups with or without large vascular lesions.

## Methods

### Participants

The Shunyi study is an ongoing prospective population-based cohort designed to investigate the risk factors and consequences of brain changes in community-dwelling adults in a Chinese population (15). All inhabitants aged 35 years or older and independently living in 5 villages of Shunyi, a suburb of Beijing, were invited to participate in this cohort study. Between June 2013 and April 2016, a total of 1,787 participants agreed to join and completed standard baseline assessments that included structured questionnaires, physical examination, and laboratory tests. All participants were invited to have brain MRI examinations. Among those, 464 refused or had contradictions to MRI, leaving 1,323 participants who underwent brain MRI. The study was approved by the Ethics Committee at Peking Union Medical College Hospital (Reference number: B-160). Written informed consent was obtained from all participants.

Of 1,323 participants who underwent brain MRI, those with poor quality images, missing urinary ACR or serum creatinine data (*n* = 333) were excluded, and subjects with a history of stroke (*n* = 62) were further excluded. Thus, the final analysis was performed based on 928 subjects. The baseline characteristics of participants included and not included in the current study were balanced.

### Vascular risk factors

Blood pressure was measured 3 times and the mean value of the 3 readings was used. Hypertension was defined as self-reported hypertension, treatment with antihypertensive medication, systolic blood pressure ≥140 mmHg, or diastolic blood pressure ≥90 mmHg. Diabetes mellitus was defined as self-reported diabetes, use of oral antidiabetic drugs or insulin, or fasting serum glucose ≥7.0 mmol/L. Hyperlipidemia was defined as self-reported hyperlipidemia, treatment with antihyperlipidemia medication, total cholesterol >5.2 mmol/L, or low-density lipoprotein >3.36 mmol/L. Smoking status was classified as current smoker (at least within the prior 1 month) and non-current smoker.

### Kidney injury measurements

Participants were asked to collect timed overnight urine samples. ACR was calculated by dividing albumin (mg) by creatinine (mmol). The ACR was naturally log-transformed because of the skewed distribution. Venous blood samples were routinely drawn after an overnight fast and an enzymatic assay method was measured for serum creatinine. eGFR was calculated based on serum creatinine using the Chronic Kidney Disease Epidemiology Collaboration formula (16).

### Magnetic resonance imaging

Participants were scanned MRI on a 3-T Skyra scanner (Siemens, Erlangen, Germany). We performed 3D T1-weighted, T2-weighted, and fluid-attenuated inversion recovery (FLAIR), and susceptibility-weighted imaging (SWI), as described in detail before (15).

All imaging markers of CSVD were defined according to the Standards for Reporting Vascular Changes on Neuroimaging. Lacunes were defined as focal lesions ranging from 3 to 15 mm in size with the same signal characteristics as cerebrospinal fluid (CSF) on all sequences situated in basal ganglia (BG) or white matter (WM). CMBs were defined as small, round, or ovoid hypointense lesions on SWI (17). Severity of dilated PVS in the BG (PVS-BG) and WM (PVS-WM) was rated using a previously established 4-level severity score (18). White matter lesions were automatically generated by using the lesion growth algorithm (19) as implemented in the Lesion Segmentation Tool (https://www.appliedstatistics.de/lst.html) for SPM. The algorithm combines segmented T1 images and co-registered FLAIR images in order to calculate the accurate lesion relief maps in white matter.

Three well-trained readers who were blinded to all clinical data rated lacunes, CMBs, and PVS independently (FH for lacunes, WQ for CMBs, SYC for PVS). Intra-rater agreement was assessed in a random sample of 50 individuals with an interval of longer than 1 month between the first and second readings. Kappa values for the intra-rater agreements were 0.73 for lacunes, 0.90 for CMBs, 0.71 for PVS-BG, and 0.61 for PVS-WM.

### Measurement of brachial-ankle pulse wave velocity and carotid plaque

Brachial-ankle pulse wave velocity (baPWV) was measured using an oscillometric method (VP-1000, Colin, Komaki, Japan) (20). In brief, the device simultaneously recorded pulse waves from the brachial and tibial arteries. The distance between each sampling point and the heart was estimated automatically according to the subject's height. The time interval between the brachium and ankle was defined as the time difference between the waveform of the brachium and waveform of the ankle. The average baPWV obtained on both sides of subjects was used for further analysis.

Ultrasound measurements were performed with a B-mode system according to a standardized scanning protocol. Plaque was defined as localized echo structures encroaching into the vessel lumen for which the distance between the media-adventitia interface and internal side of the lesion was ≥1 mm, on common carotid arteries (CCAs), carotid bifurcations, and internal carotid arteries (21).

### Statistical analyses

eGFR and Log-transformed urinary ACR were modeled continuously per SD increase. White matter hyperintensity (WMH) volume was natural log-transformed to meet normal distribution. Lacunes and microbleeds were dichotomized (present vs. absent). According to the presumed etiological background, cerebral microbleeds were categorized by location into strictly lobar vs. deep or infratentorial (with or without the presence of lobar microbleeds). Degrees of PVS in BG and WM were dichotomized into severe (degree 3 and degree 4) and mild (degree 1 and degree 2). Continuous variables are presented as mean (SD) or median (interquartile range) and categorical variables are presented as frequency and percentage. Multivariable linear regressions were used to calculate mean differences in WMH volumes and brain parenchymal fraction (BPF) for every SD increase in kidney injury measurements. Logistic regression was used to investigate the associations of kidney injury measurements with the presence of lacunes, microbleeds, and severe PVS. All models were adjusted for age and gender (model 1), and additionally for vascular risk factors including body mass index (BMI), hypertension, diabetes mellitus, hyperlipidemia, and smoking status and a history of coronary artery disease (model 2). We also performed regression models adjusted for vascular risk factors and large vessel disease markers (model 3 and model 4). All analyses involving WMH volume were adjusted for intracranial volume. To examine the modifying effect of large-vessel disease markers on the association between kidney injury measurements and CSVD MRI markers, interaction terms were added into the regression models. Stratified analyses according to carotid plaque (present or absent) and baPWV (divided equally in half) were also conducted when interactions existed. A 2-sided *P*-value of <0.05 was considered statistically significant. All analyses were performed using IBM SPSS version 23.0.

## Results

### Study population characteristics

Characteristics of the study population are shown in Table 1. Of the 928 participants, 36.1% were men and mean age was 56.0 years (SD 9.5). The median ACR was 1.11 g/mol (range 0.23–278.77), and mean eGFR was 93.5 ml/min/1.73 m^2^ (SD 14) estimated by EPI-eGFR formula. The median WMH volume was 0.97 ml (range 0–49.56), 15.1% of the participants had one or more lacunes, and 11.2% had one or more microbleeds. The median baPWV was 1,519 cm/s (range 849.5–3,360), and 43.5% of the participants had one or more carotid plaques.

**Table 1:** Characteristics of the study population.

**Characteristics**	**Mean (*SD*) or *n* (%)**
Age, years	56 (9.5)
Men, *n* (%)	335 (36.1)
BMI, kg/m^2^	26.5 (3.8)
Hypertension, *n* (%)	472 (50.9)
Current smoking, *n* (%)	204 (22.0)
Diabetes mellitus, *n* (%)	150 (16.2)
Hyperlipidemia, *n* (%)	439 (47.3)
ACR, mg/mmol^*^	1.1 (0.23–278.8)
Creatinine-based eGFR, ml/min/1.73 m^2^	93.5 (14.1)
WMHV, ml^*^	0.97 (0.0–49.6)
Lacunes, *n* (%)	140 (15.1)
Microbleeds, *n* (%)	104 (11.2)
Strictly lobar microbleeds, *n* (%)	49 (5.3)
Deep or infratentorial microbleeds, *n* (%)	55 (5.9)
PVS in basal ganglia (degree 3 or 4), *n* (%)	129 (14.4)
PVS in white matter (degree 3 or 4), *n* (%)	134 (14.7)
BPF (%)	76.3 (3.2)
baPWV, cm/s^*^	1,519 (849.5–3360)
Carotid plaques, *n* (%)	404 (43.5)

*ACR, albumin-to-creatinine ratio in urine; BMI, body mass index; eGFR, estimated glomerular filtration rate; baPWV, brachial-ankle pulse wave velocity; WMHV, White matter hyperintensity volume; PVS, perivascular space; BPF, brain parenchymal fraction, SD standardized deviation*.

* *ACR, WMHV, and baPWV were presented as median (range)*.

### Associations between kidney injury measurements and CSVD MRI markers

The associations between kidney injury measurements and MRI markers of CSVD are shown in Table 2. Participants with higher ACR had more WMH volume (adjusted mean difference for log-WMHV per SD increase of ACR: 0.11 ± 0.05, *p* = 0.034) and a higher risk of lacunes (odds ratio [OR] per SD increase of ACR, 1.27; 95% confidence interval [CI], 1.05–1.54, *p* = 0.014), while eGFR was associated with brain atrophy (adjusted mean difference in BPF per SD increase of eGFR: 0.24 ± 0.09, *p* = 0.004), risk of having lacunes (OR per SD increase of eGFR, 0.77; 95% CI, 0.61–0.96, *p* = 0.023), and risk of having deep or infratentorial microbleeds (OR per SD increase of eGFR, 0.78; 95% CI, 0.57–1.05, *p* = 0.010) (Supplemental Table 1). No association was found between PVS and kidney injury markers in basal ganglia or in white matter (Supplemental Table 2). Additional adjustment for carotid plaques or baPWV only minimally changed the associations, which remained statistically significant (Table 2).

**Table 2:** Associations between kidney injury measurements and MRI markers of CSVD.

	**log-WMHV**		**Lacunes**		**BPF**	
	**β ± *SE***	***p***	**OR (95% CI)**	***p***	**β ± *SE***	***p***
**Log-ACR**						
Model 1	0.16 ± 0.05	**0.001**	1.43(1.19,1.70)	**0.000**	−0.02 ± 0.07	0.785
Model 2	0.11 ± 0.05	**0.034**	1.27 (1.05, 1.54)	**0.014**	−0.03 ± 0.07	0.705
Model 3	0.06 ± 0.05	0.279	1.28 (1.04, 1.58)	0.018	−0.09 ± 0.08	0.597
Model 4	0.13 ± 0.05	0.017	1.35 (1.10, 1.66)	0.004	−0.05 ± 0.08	0.551
**eGFR**						
Model 1	−0.03 ± 0.06	0.578	0.77(0.62,0.96)	**0.017**	0.24 ± 0.09	**0.009**
Model 2	−0.03 ± 0.06	0.595	0.77(0.61, 0.96)	**0.023**	0.27 ± 0.09	**0.003**
Model 3	−0.02 ± 0.06	0.809	0.78 (0.62, 0.99)	0.040	0.26 ± 0.10	0.007
Model 4	−0.06 ± 0.07	0.404	0.74 (0.58, 0.94)	0.014	0.33 ± 0.10	0.001

*Model 1: adjustment for age and sex*.

*Model 2: as in Model 1, with additional adjustment for vascular risk factors (hypertension, diabetes, hyperlipidemia, current smoking, and BMI)*.

*Model 3 and Model 4: as in Model 2, with additional adjustment for baPWV and carotid plaque separately. Analyses involving white matter hyperintensity volume were additionally adjusted for intracranial volume*.

*ACR, albumin-to-creatinine ratio in urine; eGFR, estimated glomerular filtration rate; WMHV, white matter hyperintensity volume; BPF, brain parenchymal fraction*.

### Effect modification of large-vessel disease on the associations between kidney injury measurements and CSVD MRI markers

**Figure 1:**
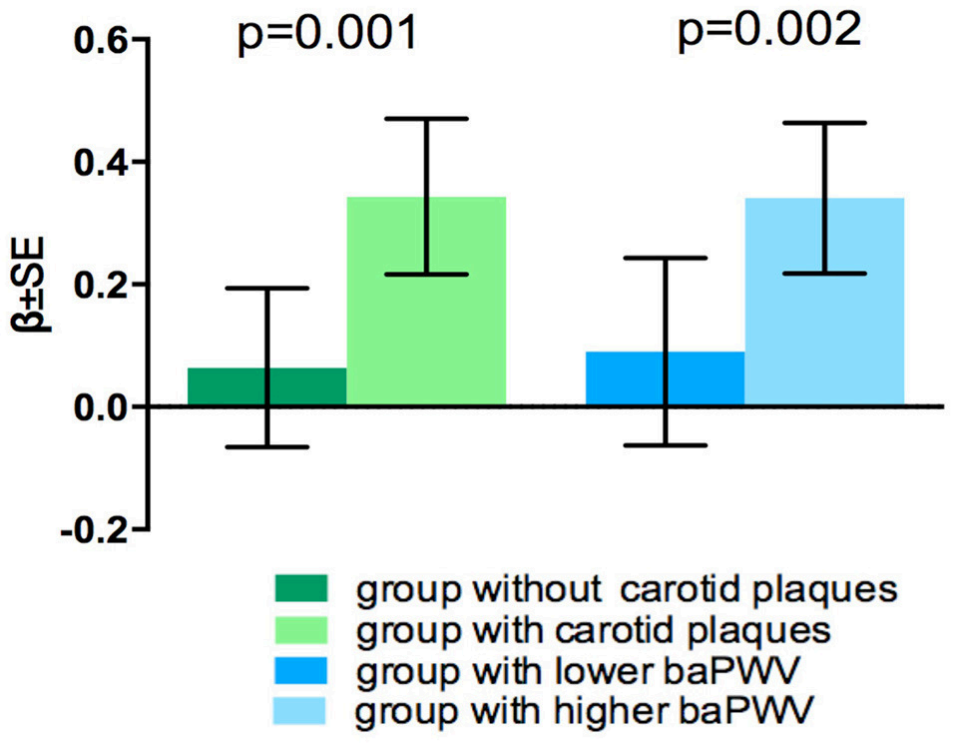
Effect modification of carotid plaques and baPWV on association between eGFR and BPF.

Coefficients and *P*-values of the interaction models are presented in Table 3. There interaction between ACR and the presence of carotid plaques on WMHV was significant (*p* = 0.035), as well as interaction between ACR and baPWV on the presence of lacunes (*p* = 0.027). Consistent significant interaction between 2 large vessel disease markers and eGFR on brain atrophy were detected (*p* = 0.001 and *p* = 0.002 respectively) (Table 3). There was no significant interaction between 2 kidney injury measurements and large-vessel parameters on CMB (lobar or deep) or PVS. In stratified analysis, the association of eGFR with BPF was only significant among participants with carotid plaques and in individuals with higher baPWV (Figure 1), but not in those without these factors. Significant association between ACR and presence of lacunes only exist in the higher baPWV group, and ACR was only significantly associated with log-WMHV in the group with carotid plaques (Supplementary Figure 1).

**Table 3:** Interactions between Carotid plaques, BaPWV, Kidney injury measurements, and CSVD MRI Markers.

	**log-WMH**		**lacunes**		**BPF**		**CMBs**	
	**Beta (std.)**	***p***	**OR (95% CI)**	***p***	**Beta (std.)**	***p***	**OR (95% CI)**	***p***
**MODEL 1**								
ACR	−0.062	0.643	4.18 (1.45, 12.08)	0.008	0.138	0.261	1.43 (0.46, 4.44)	0.538
baPWV	0.130	0.000	1.00 (1.00, 1.01)	0.051	−0.053	0.096	1.00 (1.00, 1.01)	0.301
ACR × baPWV	0.096	0.477	0.99 (0.99, 1.00)	**0.027**	−0.161	0.193	1.00 (0.99, 1.01)	0.688
**MODEL 2**								
ACR	0.001	0.985	1.56 (1.06, 2.29)	0.023	−0.046	0.222	1.03 (0.65, 1.63)	0.908
carotid plaque	0.063	0.054	2.08 (1.22, 3.55)	0.007	−0.103	0.000	1.42 (0.80, 2.51)	0.226
ACR × carotid plaque	0.091	**0.035**	0.82 (0.53, 1.29)	0.391	0.024	0.521	1.14 (0.68.1.94)	0.616
**MODEL 3**								
eGFR	−0.060	0.646	0.26 (0.09, 0.79)	0.017	−0.274	0.023	0.60 (0.20, 1.84)	0.373
baPWV	0.144	0.000	1.01 (1.01, 1.02)	0.012	−0.034	0.284	1.01 (1.00.1.01)	0.201
eGFR × baPWV	0.054	0.681	1.01 (1.00, 1.01)	0.047	0.363	**0.002**	1.00 (1.00, 1.01)	0.559
**Model 4**								
eGFR	−0.002	0.722	0.69 (0.44, 1.07)	0.095	−0.10	0.825	0.83 (0.53, 1.29)	0.397
carotid plaque	0.063	0.053	2.12 (1.25, 3.60)	0.005	−0.114	0.000	1.42 (0.81, 2.51)	0.221
eGFR × carotid plaque	−0.013	0.766	1.10 (0.68, 1.79)	0.698	0.136	**0.001**	0.98 (0.60, 1.61)	0.943

*Model 1, Model 2, Model 3, Model 4 were adjusted for age, sex and vascular risk factors (hypertension, diabetes, hyperlipidemia, current smoking, and BMI). Analyses involving white matter hyperintensity volume were additionally adjusted for intracranial volume*.

*ACR, albumin-to-creatinine ratio in urine; eGFR, estimated glomerular filtration rate; WMHV, white matter hyperintensity volume; BPF, brain parenchymal fraction*.

## Discussion

In this population-based study, we found that kidney injury measurements were associated with CSVD MRI markers independent of traditional vascular risk factors. Interaction analysis revealed that large vessel disease modified the association between decreased eGFR and brain atrophy as well as the association between increased ACR and WMHV or the presence of lacunes, while no interaction was found between large vessel disease and kidney injury measurements on CMBs or severe PVS.

Recent population-based studies have reported that both increased ACR and decreased eGFR were significantly associated with higher presence of lacunes (6, 7), which is consistent with our findings. Similarly, Our finding that lower eGFR was associated with brain atrophy was in agreement with other studies in the general population (5, 22). However, in contrast to previous literature (3, 6, 7), our study did not detect an association between decreased eGFR and log-WMHV, possibly because the lower proportion with decreased eGFR in our population attributing to a lack of statistical power. Recent studies of Korean (23) and Western populations (7) reported conflicting results for the association between impaired kidney function and CMBs. We found that decreased eGFR was associated with only deep or infratentorial CMBs, in agreement with the Korean study. A higher proportion of subjects with deep or infratentorial CMBs in Asian populations than in Western population can explain the difference. The pathophysiological mechanism underlying the cerebrorenal association is unknown. One hypothesis is that kidney injury and CSVD could be signs of systemic small vessel disease that affects different end organs (24). In addition, endothelial dysfunction, inflammatory processes, uremic toxins, sodium and water retention, and abnormal calcium and phosphate metabolism also contribute to the cerebrorenal interaction (8, 9). Interestingly, we found that increased ACR was significantly associated with ischemic MRI markers (WMHV and presence of lacunes), while decreased eGFR was significantly associated with brain atrophy. We surmise that ACR is a sensitive marker of early-stage renal disease, while decreased eGFR and brain atrophy both indicate advanced renal and cerebral small vessel lesions.

We found that the association between decreased eGFR and brain atrophy was prominent only in individuals with carotid plaque or higher baPWV, but not in those without these factors. These results provided evidence that large artery atherosclerosis or aortic stiffness modify the link between kidney injury and CSVD. The mechanism underlying the effect modification of large vessel disease is unclear, and several hypotheses have been proposed. Firstly, since both carotid plaque and increased baPWV reflect burdens of vascular risk factors and exposure duration, their accumulating effect may explain the enhanced association between kidney injury and CSVD. Secondly, large artery atherosclerosis and stiffness represent underlying endothelial dysfunction and inflammatory processes, which have been proven to play a role in small vessel disease affecting both kidney and brain (25–27). Thirdly, advanced renal disease as indicated by decreased eGFR may cause metabolic dysfunction or toxicity, further damaging large arteries and cerebral small vessels (8, 9). Fourthly, arterial stiffness can cause structural changes of blood vessels facilitating the transmission of excessive pulsatile energy into the cerebral and renal microcirculation, resulting in microvascular damage both in brain and kidney (28–30).

We also found that the association between increased ACR and WMHV was modified in the presence of carotid plaques. In addition, the association between increased ACR and presence of lacunes was affected by higher baPWV. These findings further demonstrate the impact of large artery changes on the cerebrorenal interaction, and suggest that this effect may be apparent in individuals with early stage renal disease.

The strength of our study includes the large sample size, use of both ACR and eGFR as kidney injury measurements, quantitative measurement of CSVD MRI markers, and simultaneous assessment of large artery atherosclerosis and stiffness. There are also several limitations. First, ACR and serum creatinine were only measured once and this could bias our results. Second, although we adjusted for important and potential vascular risk factors in the analysis of the association between kidney injury measurements and CSVD and effect modification of large-vessel disease, we cannot rule out residual unmeasured confounders. Third, the analysis was cross-sectional and we cannot draw conclusions of causality.

## Conclusion

Kidney injury was independently associated with CSVD, and both large artery atherosclerosis and stiffness enhanced the association between renal disease and CSVD. Our findings provided evidence of effect modification of large-vessel disease on the association between kidney injury and CSVD. Evaluation of subclinical CVSD in individuals with kidney injury is warranted, especially in those with combined large artery disease. Further studies on the mechanisms of this association are needed.

## Author contributions

D-HA drafted the manuscript and conducted the statistical analyses. F-FZ managed the database. FH, L-XZ, JN, MY, D-DZ, M-LL, X-HF, Z-YJ, L-YC, and S-YZ contributed to the study design, data collection and interpretation. All authors approved for the manuscript submitted for publication. Y-CZ was the principal investigator of the study and responsible for the decision to submit for publication.

### Conflict of interest statement

The authors declare that the research was conducted in the absence of any commercial or financial relationships that could be construed as a potential conflict of interest.
