# Supplementary material for: Large Vessel Disease Modifies the Relationship Between Kidney Injury and Cerebral Small Vessel Disease
Source: Front Neurol. 2018 Jun 26;9:498. doi: 10.3389/fneur.2018.00498 (PMC6028610; doi:10.3389/fneur.2018.00498)
Supplement: Supplementary file 1 [file Presentation_1.pdf]

Supplemental Table 1[a]

	CMB		Strictly Lober CMB		Deep CMB	
	OR(95%CI)	*P*	OR(95%CI)	*P*	OR(95%CI)	*P*
ACR						
Model 1	1.15 (0.93, 1.42)	0.191	1.06 (0.77,1.40)	0.824	1.26 (0.98,1.63)	0.069
Model 2	1.16 (0.96, 1.41)	0.120	1.05 (0.75, 1.38)	0.770	1.22 (0.94, 1.58)	0.142
Model 3	1.14 (0.91, 1.43)	0.272	1.02 (0.73, 1.43)	0.897	1.22 (0.91, 1.63)	0.179
Model 4	1.13 (0.90, 1.44)	0.293	0.99 (0.69, 1.42)	0.956	1.25 (0.94, 1.68)	0.129
eGFR						
Model 1	0.85 (0.67, 1.07)	0.169	0.99 (0.70,1.41)	0.955	0.76 (0.57,1.02)	0.066
Model 2	0.85 (0.67, 1.08)	0.195	0.99 (0.69, 1.40)	0.933	0.78 (0.57, 1.05)	0.010
Model 3	0.83 (0.65, 1.07)	0.153	0.99 (0.69, 1.42)	0.934	0.74 (0.54, 1.02)	0.065
Model 4	0.82 (0.63, 1.05)	0.119	0.87 (0.60, 1.26)	0.458	0.78 (0.57, 1.08)	0.133

[a]Model 1: adjustment for age and sex; Model 2: as in Model 1, with additional adjustment for vascular risk factors (hypertension, diabetes, hyperlipidemia, current smoking, and BMI); Model 3 and Model 4: as in Model 2, with additional adjustment for carotid plaque and baPWV separately.

ACR, albumin-to-creatinine ratio in urine; eGFR, estimated glomerular filtration rate; CMB cerebral microbleed

Supplement Table 2[a]

	Basel ganglia PVS		White matter PVS		SVD score	
	β（95%CI）	*P*	OR(95%CI)	*P*	β±SE	p
ACR						
Model 1	1.08 (0.90, 1.31)	0.416	1.10 (0.92,1.32)	0.309	0.088±0.026	0.001
Model 2	1.12 (0.92, 1.36)	0.263	1.10 (0.91, 1.33)	0.311	0.074±0.027	0.005
Model 3	1.14 (0.92, 1.40)	0.234	1.10 (0.89, 1.34)	0.389	0.071±0.029	0.014
Model 4	1.15 (0.92, 1.42)	0.217	1.11 (0.90, 1.36)	0.329	0.087±0.029	0.003
eGFR						
Model 1	1.00 (0.80,1.25)	0.979	1.18 (0.93, 1.51)	0.174	-0.073±0.030	0.015
Model 2	1.00 (0.80, 1.26)	0.993	1.26 (0.95, 1.58)	0.114	-0.076±0.030	0.013
Model 3	0.94 (0.74, 1.19)	0.596	1.17 (0.90, 1.50)	0.239	-0.08±0.032	0.013
Model 4	1.03 (0.79, 1.33)	0.846	1.14 (0.87, 1.49)	0.343	-0.083±0.035	0.017

[a]Model 1: adjustment for age and sex; Model 2: as in Model 1, with additional adjustment for vascular risk factors (hypertension, diabetes, hyperlipidemia, current smoking, and BMI); Model 3 and Model 4: as in Model 2, with additional adjustment for carotid plaque and baPWV separately.

ACR, albumin-to-creatinine ratio in urine; eGFR, estimated glomerular filtration rate; PVS perivascular space

Supplementary figure 1

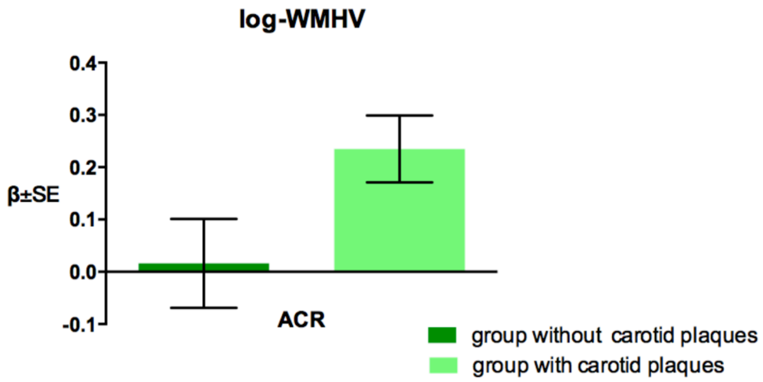

A. Effect modification of carotid plaques on association between ACR and WMHV.

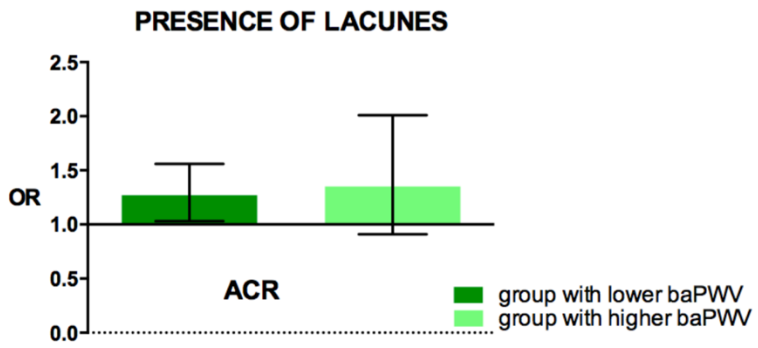

B. Effect modification of baPWV on association between ACR and lacunes.
